# Morpho-phylogenetic analyses reveal *Dentisympodium fusiforme* gen. et sp. nov. in Pleurotheciaceae (Pleurotheciales, Savoryellomycetidae) from China

**DOI:** 10.3897/mycokeys.136.195305

**Published:** 2026-07-06

**Authors:** Wangming Zhang, Qinying Feng, Wanqing Xie, Xiaoyu Song, Xinzhong Zhou, Juan Lu, Yuxi Yang, Zhongzhe Li

**Affiliations:** 1 Beijing Jishuitan Hospital Guizhou Hospital, Guiyang 550014, China Beijing Jishuitan Hospital Guizhou Hospital Guiyang China

**Keywords:** freshwater fungi, hyphomycetes, phylogeny, Sordariomycetes, taxonomy

## Abstract

Pleurotheciaceae is a diverse family of hyphomycetous fungi within Pleurotheciales (Savoryellomycetidae), comprising numerous saprobic species that contribute to the decomposition of organic matter in freshwater ecosystems. Although the family has undergone considerable taxonomic expansion in recent years, its diversity remains insufficiently documented, particularly in freshwater habitats, where novel lineages continue to be discovered. During investigations of freshwater fungi associated with submerged decaying wood in Guizhou Province, China, fungal specimens were collected and examined using morphological observations and multigene phylogenetic analyses. Phylogenetic relationships were inferred from a concatenated dataset comprising the ITS, LSU, SSU, and *rpb*2 loci, and morphological characteristics were compared with those of related genera within Pleurotheciaceae. The results revealed a distinct lineage within Pleurotheciaceae, for which the new genus *Dentisympodium* is introduced to accommodate the new species *D. fusiforme*. Phylogenetic analyses placed *Dentisympodium* within Pleurotheciaceae with strong statistical support. The genus is currently known only from its asexual morph and is characterized by macronematous, mononematous, erect, unbranched, subcylindrical, flexuous, hyaline conidiophores; holoblastic, polyblastic, integrated, terminal, sympodial, irregularly subcylindrical conidiogenous cells bearing denticles; and solitary, acropleurogenous, predominantly 3-septate, cylindrical, fusiform to obclavate, hyaline conidia. Morphological comparisons demonstrated that *Dentisympodium* is readily distinguishable from other genera of Pleurotheciaceae, particularly by its pseudodactylaria-like conidia and unique conidiogenous apparatus. The introduction of *Dentisympodium* expands the morphological and phylogenetic diversity currently recognized within Pleurotheciaceae and highlights the importance of continued exploration of freshwater fungi for uncovering previously unknown fungal taxa and refining the taxonomy of the family.

## Introduction

Lignicolous freshwater fungi are a morphologically diverse group of fungi that inhabit a wide range of freshwater environments, including streams, rivers, lakes, and reservoirs (Luo et al. 2004; Hyde et al. 2016; Calabon et al. 2022, 2023). These fungi are crucial decomposers of lignocellulosic substrates, mediating organic matter turnover and nutrient cycling, thereby maintaining ecosystem stability and supporting freshwater biodiversity (Yuen et al. 1998; Bucher et al. 2004; Calabon et al. 2023). Over the past few decades, Asia has emerged as a major hotspot for research on lignicolous freshwater fungi, with studies primarily focusing on their diversity and taxonomy (Su et al. 2016; Luo et al. 2019; Dong et al. 2020; Bao et al. 2021, 2023; Shen et al. 2024, 2025). Significant progress has been achieved, particularly in regions of China (including Guangxi, Guizhou, Hainan, Hong Kong, Taiwan, and Yunnan) and Thailand (Chiang Rai and Chiang Mai) (Zhang et al. 2011; Jones et al. 2014; Bao et al. 2018, 2019, 2020; Luo et al. 2018a, 2018b; Calabon et al. 2021; Dong et al. 2021a, 2021b; Hyde et al. 2021). The discovery of numerous novel taxa has greatly expanded our understanding of the diversity and distribution patterns of these fungi in tropical and subtropical regions (Shen et al. 2022a, 2022b; Xu et al. 2023, 2024b, 2025a, 2025b; Wang et al. 2024a, 2024b, 2025; Xiao et al. 2025a).

Pleurotheciaceae is one of the largest families within the subclass Savoryellomycetidae and comprises a diverse group of predominantly saprobic fungi that occur in freshwater and terrestrial habitats worldwide (Réblová et al. 2016, 2020; Hyde et al. 2020; Kuo et al. 2024). The family Pleurotheciaceae Réblová & Seifert was established by Réblová et al. (2016), with *Pleurothecium* designated as the type genus. Members of the family exhibit considerable morphological diversity in their asexual morphs, which include acrodictys-like, helicoön-like, monodictys-like, and dactylaria-like forms (Hyde and Hyde 2002; Sadowski et al. 2012; Réblová et al. 2016, 2020; Luo et al. 2018a; Dayarathne et al. 2019; Hyde et al. 2020; Kuo et al. 2024). The sexual morph is characterized by dark, papillate, perithecial, astromatic, immersed to superficial ascomata, unitunicate asci with a distinct non-amyloid apical annulus, and fusiform to ellipsoidal, septate, hyaline ascospores (Réblová et al. 2016; Luo et al. 2018a; Hyde et al. 2020). Currently, the family Pleurotheciaceae comprises 17 accepted genera, including *Adelosphaeria* Réblová, *Anapleurothecium* Hern.-Restr., R.F. Castañeda & Gené, *Coleodictyospora* Charles, *Dematipyriforma* L. Yan Sun, Hai Y. Li, Xiang Sun & L.D. Guo, *Helicoascotaiwania* Dayar., Maharachch. & K.D. Hyde, *Melanotrigonum* Réblová, *Monotosporella* S. Hughes, *Neomonodictys*, Y.Z. Lu, C.G. Lin & K.D. Hyde, *Nigrellomyces* Wang M. Zhang & Q.Y. Feng, *Phaeoisaria* Höhn., *Phragmocephala* E.W. Mason & S. Hughes, *Pleurotheciella* Réblová, Seifert & J. Fourn, *Pleurothecium* Höhn., *Pseudosaprodesmium* X.G. Tian, K.D. Hyde & Tibpromma, *Rhexoacrodictys* W.A. Baker & Morgan-Jones, *Saprodesmium* W. Dong & Doilom, and *Sterigmatobotrys* Oudem (Xia et al. 2017; Hyde et al. 2024; Zhang et al. 2025). Despite recent advances, several aspects of Pleurotheciaceae taxonomy remain insufficiently understood. Molecular sequence data are still unavailable for numerous taxa, several genera are represented by only a limited number of collections, and the phylogenetic placement of some morphologically similar taxa remains unresolved. Furthermore, freshwater habitats have proven to be important reservoirs of pleurotheciaceous diversity, and ongoing surveys continue to reveal previously undocumented lineages and novel taxa (Zhang et al. 2011, 2025; Réblová et al. 2016; Luo et al. 2018a; Dong et al. 2021b; Shi et al. 2021; Bao et al. 2022; Huang et al. 2022; Wang et al. 2024a; Shen et al. 2025). These findings suggest that the species diversity, phylogenetic relationships, and generic circumscriptions within Pleurotheciaceae remain incompletely resolved and warrant further investigation.

During a recent survey of lignicolous freshwater fungi in Guizhou Province, China, we identified a previously unrecognized taxon resembling *Pseudodactylaria*. Multi-locus phylogenetic analyses of the combined ITS, LSU, SSU, and *rpb*2 sequence dataset revealed that the newly collected isolates form a well-supported, distinct lineage within Pleurotheciaceae. Integrating detailed morphological observations with robust phylogenetic evidence, we herein propose a novel genus to accommodate this unique lineage. The study presents a comprehensive taxonomic treatment, including precise morphological descriptions, high-resolution illustrations, and comparative analyses with phylogenetically related taxa.

## Materials and methods

### Sample collection and specimen examination

Fresh specimens of decaying wood were collected from a freshwater stream in Baiyun District, Guiyang City, Guizhou Province, China, on 12 October 2025. Samples were taken to the laboratory in plastic bags, labeled with collection details, including locality, habitat and date (Rathnayaka et al. 2024). Samples were cultured in plastic boxes lined with moistened tissue at room temperature for 1–2 weeks (Yang et al. 2023). The samples were examined using a stereomicroscope (SMZ 800, Nikon, Japan). Micro-morphological characters were captured using an EOS 90D digital camera attached to an ECLIPSE Ni compound microscope (Nikon, Japan). Measurements of conidiophores, conidiogenous cells, and conidia were carried out using the Tarosoft (R) Image Frame Work program.

### Isolation and material deposition

Single-spore isolation was performed following the method described by Senanayake et al. (2020). The germinated conidia were aseptically transferred to fresh potato dextrose agar (PDA; Oxoid, CM0139) and incubated at room temperature for 36 days. Morphological characteristics of the fungal mycelium on PDA, including color, shape, size, margin, and elevation, were documented. Dried fungal specimens were deposited in the Herbarium of Guizhou Academy of Agriculture Sciences (Herb. GZAAS), Guiyang, China. Pure cultures were deposited at the Guizhou Culture Collection (GZCC), Guiyang, China. Descriptions of the new taxa were uploaded to the Faces of Fungi webpage following the guidelines of Jayasiri et al. (2015). The new species were registered in the MycoBank database (https://www.mycobank.org/), and MycoBank numbers were obtained.

### DNA extraction, PCR amplification, and sequencing

The mycelium, freshly scraped from living cultures, was transferred to 1.5 mL microcentrifuge tubes, and stored in a refrigerator at -20 °C. Genomic DNA was extracted using the Biospin Fungus Genomic DNA Extraction Kit (BioFlux, China), following the manufacturer’s protocol. The primer pairs ITS5/ITS4 (White et al. 1990), LR0R/LR5 (Vilgalys and Hester 1990), NS1/NS4 (White et al. 1990), and fRPB2-5F/fRPB2-7cR (Liu et al. 1999) were used to amplify the ITS, LSU, SSU, and *rpb*2 regions, respectively. PCR amplification was performed in a 25 μL reaction volume, consisting of 13.5 μL of 2× Taq PCR Master Mix (China; containing Taq DNA polymerase, dNTPs, MgCl_2_, and reaction buffer), 1 μL of each primer, 1 μL of template DNA, and 8.5 μL of ddH_2_O. The polymerase chain reaction (PCR) conditions employed were in accordance with the reaction conditions outlined in the publications by Zhang et al. (2025). The PCR products were purified and sequenced by Sangon Biotech (Shanghai, China) Co., Ltd.

### Phylogenetic analyses

The forward and reverse primers of the newly generated sequences were checked and assembled using BioEdit v. 7.0.5.3 (Hall 1999) and SeqMan v. 7.0.0 (Swindell and Plasterer 1997). Similar taxa were identified through BLASTn searches conducted on the NCBI website (Table 1, https://blast.ncbi.nlm.nih.gov/Blast.cgi). Multiple sequence alignments for each locus dataset were performed using MAFFT v.7.473 (https://mafft.cbrc.jp/alignment/server/, Katoh et al. 2019) and visually inspected in AliView (Larsson 2014). The ITS, LSU, SSU, and *rpb*2 alignments were trimmed using trimAl v1.2rev59 (Capella-Gutiérrez et al. 2009), and subsequently merged using SequenceMatrix v1.7.8 (Vaidya et al. 2011).

**Table 1.** Taxa used in this study, along with their corresponding GenBank accession numbers.

**Taxon**	**Strain/Specimens**	**GenBank Accession Numbers**				**References**
		**ITS**	**LSU**	**SSU**	***rpb*2**	
*Adelosphaeria catenata*	CBS 138679^T^	KT278721	KT278707	KT278692	KT278743	Réblová et al. (2016)
*Anapleurothecium botulisporum*	FMR 11490^T^	KY853423	KY853483	-	-	Hernández-Restrepo et al. (2017)
*Ascotaiwania fusiformis*	MFLU 15-1156^T^	MG388215	NG_057114	-	KX576871	Yang et al. (2016)
*Ascotaiwania sawadae*	SS00051	HQ446340	HQ446363	HQ446283	HQ446418	Boonyuen et al. (2011)
*Canalisporium exiguum*	SS00809	GQ390296	GQ390281	GQ390266	HQ446436	Sri-indrasutdhi et al. (2010)
*Canalisporium grenadoideum*	SS03615	-	GQ390267	GQ390252	HQ446420	Sri-indrasutdhi et al. (2010)
*Coleodictyospora muriformis*	MFLUCC 18-1243^T^	MW981642	MW981648	MW981704	-	Dong et al. (2021b)
*Conioscypha lignicola* ^O^	CBS 335.93^T^	-	AY484513	JQ437439	JQ429260	Réblová et al. (2016)
*Conioscypha minutispora* ^O^	CBS 137253^T^	-	MH878131	-	-	Vu et al. (2019)
*Dematipyriforma aquilaria*	CGMCC 3.17268^T^	KJ138621	KJ138623	KJ138622	-	Bao et al. (2022)
*Dematipyriforma muriformis*	MFLU 21-0146^T^	OM654773	OM654770	-	-	Bao et al. (2022)
***Dentisympodium fusiforme***	**GZCC 25-27594^T^**	**PZ296709**	**PZ296717**	**-**	**-**	**In this study**
***Dentisympodium fusiforme***	**GZCC 25-27595**	**PZ296710**	**PZ296718**	**-**	**-**	**In this study**
*Helicoascotaiwania lacustris*	CBS 145963^T^	-	MN699430	-	MN704304	Réblová et al. (2020)
*Helicoascotaiwania lacustris*	CBS 145964	MN699400	MN699431	MN699383	MN704305	Réblová et al. (2020)
*Melanotrigonum ovale*	CBS 138742	KT278723	KT278708	KT278695	KT278744	Réblová et al. (2016)
*Melanotrigonum ovale*	CBS 138815	KT278722	KT278711	KT278698	KT278747	Réblová et al. (2016)
*Monotosporella setosa*	HKUCC 3713	-	AF132334	-	-	Ranghoo et al. (1999)
*Neomonodictys aquatica*	KUNCC 21-10708^T^	MZ686200	OK245417	-	-	Bao et al. (2022)
*Neomonodictys muriformis*	MFLUCC 16-1136^T^	MN644509	MN644485	-	-	Bao et al. (2022)
*Nigrellomyces aquaticus*	GZCC 25-0630^T^	PV871229	PV871235	-	PV872880	Zhang et al. (2025)
*Obliquifusoideum guttulatum*	MFLUCC 18-1233^T^	MW981645	MW981650	MW981706	-	Dong et al. (2021b)
*Obliquifusoideum triseptatum*	CGMCC 3.27014^T^	PP445243	PP049503	PP049521	PP068779	Wang et al. (2024a)
*Phaeoisaria annesophieae*	CBS 143235^T^	MG022180	MG022159	-	-	Wang et al. (2025)
*Phaeoisaria aquatica*	MFLUCC 16-1298^T^	MF399237	MF399254	-	MF401406	Luo et al. (2018a)
*Phaeoisaria dalbergiae*	CPC 39540^T^	OK664703	OK663742	OK663796	OK651159	Crous et al. (2021)
*Phaeoisaria ellipsoidea*	IFRDCC 3134^T^	ON533383	ON533387	-	-	Wang et al. (2025)
*Phaeoisaria fasciculata*	CBS 127885^T^	KT278719	KT278705	KT278693	KT278741	Réblová et al. (2016)
*Phaeoisaria filiformis*	MFLUCC 18-0214^T^	MK878381	MK835852	MK834785	-	Bao et al. (2019)
*Phaeoisaria goiasensis*	FCCUFG 02^T^	MT210320	MT375865	-	-	Jayawardena et al. (2023)
*Phaeoisaria guttulata*	MFLUCC 17-1965^T^	MG837021	MG837016	MG837026	-	Hyde et al. (2018)
*Phaeoisaria laianensis*	JAUCC 4967^T^	ON937559	ON937557	ON937562	-	Liu et al. (2022)
*Phaeoisaria loranthacearum*	CBS 140009^T^	KR611888	MH878676	-	-	Wang et al. (2025)
*Phaeoisaria microspora*	MFLUCC 16-0033^T^	MF671987	MF167351	-	MF167352	Wang et al. (2025)
*Phaeoisaria motuoensis*	KUNCC 10410^T^	OP626333	OQ947034	OQ947036	-	Xu et al. (2024a)
*Phaeoisaria obovate*	CGMCC 3.27015^T^	PP049488	PP049504	PP049522	PP068788	Wang et al. (2024a)
*Phaeoisaria pseudoclematidis*	MFLUCC 11-0393^T^	NR_155648	NG_059559	KP753962	-	Liu et al. (2015)
*Phaeoisaria sedimenticola*	CGMCC 3.14949^T^	JQ074237	JQ031561	-	-	Cheng et al. (2014)
*Phaeoisaria siamensis*	MFLUCC 16-0607^T^	MK607610	MK607613	MK607612	MK607611	Hyde et al. (2019)
*Phaeoisaria sparsa*	FMR 11939	PV455937	PV455951	PV455964	PV483451	Wang et al. (2025)
*Phaeoisaria synnematica*	NFCCI 4479^T^	MK391494	MK391492	-	-	Boonmee et al. (2021)
*Phragmocephala stemphylioides*	KAS 4277	KT278730	KT278717	-	-	Réblová et al. (2016)
*Pleurotheciella saprophytica*	MFLUCC 16-1251^T^	MF399241	MF399258	MF399224	MF401410	Luo et al. (2018a)
*Pleurotheciella submersa*	MFLUCC 17-1709^T^	MF399243	MF399260	MF399226	MF401412	Luo et al. (2018a)
*Pleurothecium pulneyense*	MFLUCC 16-1293	-	MF399262	MF399228	MF401414	Luo et al. (2018a)
*Pleurothecium semifecundum*	CBS 131271^T^	JQ429159	JQ429240	JQ429254	JQ429270	Réblová et al. (2012)
*Pseudosaprodesmium cocois*	MFLU 23-0225^T^	OR438401	OR438864	OR458363	-	Tian et al. (2024)
*Pseudosaprodesmium narathiwatense*	MFLU 24-0506^T^	PV271889	PV271928	PV263318	PV340530	Karimi et al. (2025)
*Rhexoacrodictys fimicola*	MFLUCC 18-0340	OM654774	OM654771	OM654806	-	Bao et al. (2022)
*Rhexoacrodictys melanospora*	KUNCC 22-12406^T^	OP168085	OP168087	OP168088	OP208807	Bao et al. (2023)
*Saprodesmium dematiosporium*	KUMCC 18-0059^T^	MW981646	MW981647	MW981707	-	Dong et al. (2021b)
*Sterigmatobotrys macrocarpa*	DAOM 230059	JQ429154	GU017316	-	-	Réblová et al. (2012)
*Sterigmatobotrys macrocarpa*	PRM 915682	JQ429153	GU017317	JQ429255	-	Réblová et al. (2012)
*Sterigmatobotrys rudis*	DAOM 229838	JQ429152	JQ429241	JQ429256	JQ429272	Réblová et al. (2012)

Note: “^T^” and “^O^” indicate ex-type strains and outgroup strains, respectively. Newly generated sequences are in black bold. “-” indicates the unavailable data in GenBank.

Phylogenetic analyses were conducted using RAxML-HPC v.8 on XSEDE (8.2.12), with a GTRGAMMA model and rapid bootstrap analysis followed by 1000 bootstrap replicates (Stamatakis 2014). Bayesian Inference (BI) analysis was performed using MrBayes on XSEDE (3.2.7a) via CIPRES Science Gateway (Swofford 2002; Ronquist et al. 2012). The aligned FASTA file was converted to NEXUS format using AliView (Daniel et al. 2010). The best-fit evolutionary model for each dataset was determined using MrModeltest v. 2.3. 10 (Nylander 2004). The GTR+I+G substitution model was selected for LSU, ITS, and *rpb*2, whereas the SYM+G model was chosen for SSU. The posterior probabilities (PP) were determined based on Bayesian Markov chain Monte Carlo (BMCMC) sampling (Huelsenbeck and Ronquist 2001). Four simultaneous Markov chains were run for 10,000,000 generations, and trees sampled every 1,000^th^ generation. The burn-in phase was set at 25%, and the remaining trees were used to calculate posterior probabilities.

Phylogenetic trees were visualized using FigTree v. 1.4.4 and further edited in PowerPoint. The photo-plate was made using Adobe Photoshop CS6 software (Adobe Systems, the USA).

### Phylogenetic analysis results

A multi-locus phylogenetic analysis based on combined ITS, LSU, SSU, and *rpb*2 sequence data was performed to determine the phylogenetic placement of the two novel strains. The concatenated alignment comprised 3,503 characters (ITS: 1–613, LSU: 613–1,478, SSU: 1,479–2,476, and *rpb*2: 2,477–3,534) representing 54 taxa. Phylogenetic reconstructions were conducted using maximum likelihood (ML) and Bayesian inference (BI) methods on the combined dataset, both of which produced congruent topologies. Estimated base frequencies were A = 0.234649, C = 0.261852, G = 0.292542, and T = 0.210957, while substitution rates were AC = 1.849681, AG = 3.037569, AT = 1.772392, CG = 1.499636, CT = 8.247664, and GT = 1.000000. The gamma distribution shape parameter (α) was 0.241402.

**Figure 1.**
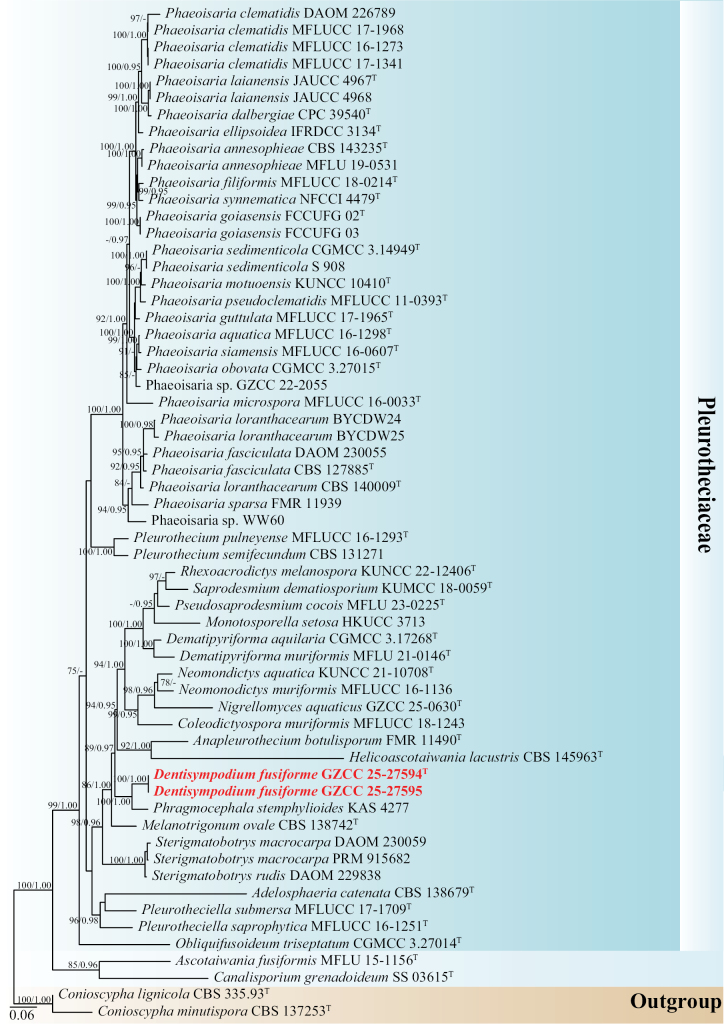
Phylogenetic tree inferred from maximum likelihood (ML) analysis based on the combined ITS, LSU, SSU, and *rpb*2 sequence dataset. Bootstrap support values ≥ 75% for ML (left) and Bayesian posterior probabilities (PP) ≥ 0.95 (right) are indicated at the nodes. Hyphen (“-”) indicates a value lower than 75% for ML and a posterior probability lower than 0.95 for BI. The tree is rooted with *Conioscypha lignicola* (CBS 335.93) and *C. minutispora* (CBS 137253). Ex-type strains are indicated by “^T^”, and newly obtained isolates are highlighted in bold red.

According to a BLASTn search on NCBI GenBank, the ITS and LSU sequences of the new isolates (GZCC 25-27594 and GZCC 25-27594) share 89.55% similarity across 92% of the query sequence coverage and 97.98% similarity across 97% of the query sequence coverage with *Phragmocephala stemphylioides* (KAS 4277), respectively. Based on the multi-gene phylogenetic reconstruction (Fig. 1), our collections are resolved as a distinct lineage within Pleurotheciaceae (Pleurotheciales, Sordariomycetes), representing a novel genus and species. The isolates GZCC 25-27594 and GZCC 25-27594 cluster as a well-supported sister clade to *Phragmocephala stemphylioides* (KAS 4277), with maximum likelihood bootstrap and Bayesian posterior probability values of 100% ML and 1.00 PP, respectively.

## Taxonomy

### Dentisympodium

Taxon classificationFungiPleurothecialesSavoryellomycetidae

W.M. Zhang & Z.Z. Li gen. nov.

7C1CA301-4B0B-593F-BB5B-9E1302AB518F

905400

#### Etymology.

“*Dentisympodium*” refers to the distinct cylindrical denticles (“denti-”) and sympodial proliferation (“sympodium”) of the conidiogenous cells.

#### Description.

***Saprobic*** on submerged decaying wood in a freshwater habitat. ***Sexual morph***: undetermined. ***Asexual morph***: hyphomycetous. ***Colonies*** on the substratum superficial, effuse, gregarious, white. ***Mycelium*** composed of partly immersed, partly superficial, hyaline, septate, branched hyphae. ***Conidiophores*** macronematous, mononematous, solitary, erect, unbranched, subcylindrical, flexuous, hyaline, 0–1-septate. ***Conidiogenous cells*** holoblastic, polyblastic, integrated, terminal, sympodial, irregular subcylindrical, flexuous, hyaline, apical part forming a rachis with numerous, aggregated, cylindrical denticles. ***Conidia*** solitary, acropleurogenous, 0–3-septate, mostly 3-septate, cylindrical, fusiform to obclavate, subobtuse at apex, hyaline, smooth, prominently guttulate.

#### Type species.

*Dentisympodium fusiforme* W.M. Zhang & Z.Z. Li.

#### Notes.

Morphologically, *Dentisympodium* can be readily distinguished from other genera in Pleurotheciaceae by its macronematous, mononematous, erect, unbranched, subcylindrical, flexuous, hyaline conidiophores; holoblastic, polyblastic, integrated, terminal, sympodial, irregularly subcylindrical, flexuous, hyaline conidiogenous cells bearing denticles; and solitary, acropleurogenous, predominantly 3-septate, cylindrical, fusiform to obclavate, hyaline conidia. Phylogenetically, *Dentisympodium* forms a distinct clade within Pleurotheciaceae, supporting its recognition as a novel genus. Accordingly, we introduce *Dentisympodium* to accommodate a new species, *D. fusiforme*, which is designated as the type species based on both molecular evidence and its distinctive conidial morphology.

### Dentisympodium fusiforme

Taxon classificationFungiPleurothecialesSavoryellomycetidae

W.M. Zhang & Z.Z. Li sp. nov.

CDCC115B-346C-505C-8FDA-27B70063E80F

905401

Fig. 2

#### Etymology.

The species epithet “*fusiforme*’’ refers to the fusiform conidia of this fungus.

#### Holotype.

GZAAS 25-0785.

#### Description.

***Saprobic*** on submerged decaying wood in a freshwater habitat. ***Sexual morph***: undetermined. ***Asexual morph***: hyphomycetous. ***Colonies*** on the substratum superficial, effuse, gregarious, white. ***Mycelium*** composed of partly immersed, partly superficial, hyaline, septate, branched hyphae. ***Conidiophores*** 17–24 × 4–5 μm (*x̄* = 19 × 4.5 μm, n = 20), macronematous, mononematous, solitary, erect, unbranched, subcylindrical, flexuous, hyaline, 0–1-septate. ***Conidiogenous cells*** 8.5–11 × 3.5–4.5 μm (*x̄* = 10 × 4 μm, n = 20), holoblastic, polyblastic, integrated, terminal, sympodial, irregular subcylindrical, flexuous, hyaline, apical part forming a rachis with numerous, aggregated, cylindrical denticles. ***Conidia*** 19–34 × 3.5–5 μm (*x̄* = 30 × 5 μm, n = 35), solitary, acropleurogenous, 0–3-septate, mostly 3-septate, cylindrical, fusiform to obclavate, subobtuse at apex, hyaline, smooth, prominently guttulate.

#### Culture characteristics.

Conidia germinate on PDA within 11 hours, producing germ tubes from the conidial body. Colonies on PDA are irregular, with a flat to slightly raised surface and an entire margin, reaching 31 cm in diameter after 36 days at room temperature (approximately 25 °C). Surface light brown to dark brown, with the point of attachment dark brown to nearly black, exhibiting radiating pigmentation; reverse pale yellow to grayish yellow, with a slightly darker center showing olivaceous to gray-green tinges; margin lighter, appearing pale yellow to whitish.

**Figure 2.**
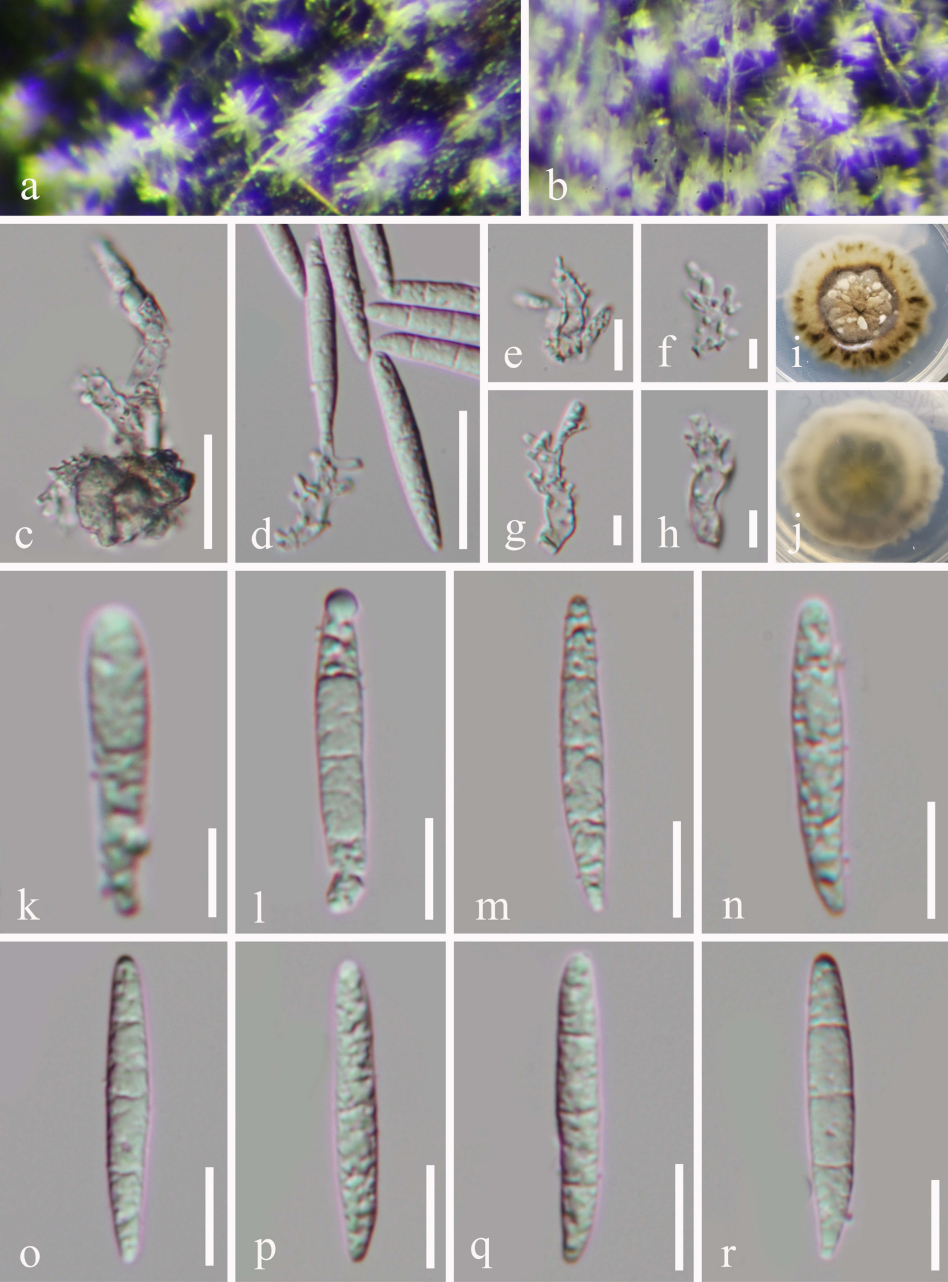
*Dentisympodium fusiforme* (GZAAS 25-0785, holotype). **a, b**. Colonies on the host surface; **c, d**. Conidiophores, conidiogenous cells, and conidia; **g**. Conidiogenous cells with attached conidium; **e, f, h**. Conidiogenous cells; **k–r**. Conidia; **i, j**. Colonies on PDA from above and below after 36 days of incubation at room temperature. Scale bars: 20 μm (**c, d**); 10 μm (**e, l–r**); 5 μm (**f–h, k**).

#### Material examined.

China • Guizhou Province, Guiyang City, Baiyun District, Changpo Ling National Forest Park, on decaying wood in a freshwater habitat, 12 October 2025, Wang-Ming Zhang & Zhongzhe Li, GZAAS 25-0785, ***holotype***, ex-type living culture GZCC 27-27594; • *ibid*., GZAASGZAAS 25-0786, ***isotype***, ex-isotype culture GZCC 27-27595.

#### Notes.

In the phylogenetic tree (Fig. 1), the isolates GZCC 27-27594 and GZCC 27-27595 form a sister clade to *Phragmocephala stemphylioides* (KAS 4277) with strong support (100% ML, 1.00 PP). However, pairwise sequence comparisons between *Dentisympodium fusiforme* (GZCC 27-27594) and *P. stemphylioides* reveal 56/536 bp differences in ITS (10.5%, including 20 gaps) and 17/842 bp differences in LSU (2.0%, excluding gaps). Morphologically, *D. fusiforme* is clearly distinguishable from *P. stemphylioides* by its hyaline conidiophores; sympodial, irregularly subcylindrical, hyaline conidiogenous cells bearing denticles; and cylindrical, fusiform to obclavate, hyaline conidia. In contrast, *P. stemphylioides* possesses pale brown to brown conidiophores, subhyaline to pale brown, cylindrical conidiogenous cells, and obovoid, dematiaceous conidia (Hughes 1958; Réblová et al. 2016; Bao et al. 2022). Therefore, based on multigene phylogenetic evidence together with distinct morphological differences, we introduce a new genus, *Dentisympodium*, to accommodate *D. fusiforme* as a novel species.

## Discussion

The newly introduced genus *Dentisympodium* exhibits morphological similarities to *Pseudodactylaria*, including macronematous, mononematous, erect, unbranched, hyaline conidiophores; holoblastic, polyblastic, integrated, terminal, sympodial, hyaline conidiogenous cells bearing denticles; and solitary, acropleurogenous, cylindrical to fusiform or obclavate, hyaline conidia (Crous et al. 2017; Hyde et al. 2020; Lu et al. 2020; Shen et al. 2025; Xiao et al. 2025a, 2025b; Xu et al. 2025a). However, *Dentisympodium* can be readily distinguished from *Pseudodactylaria* by its irregularly subcylindrical conidiophores and predominantly 3-septate conidia, whereas *Pseudodactylaria* possesses strictly cylindrical conidiophores of a length similar to those of *Dentisympodium* and 0–1-septate conidia (Crous et al. 2017; Hyde et al. 2020; Lu et al. 2020; Shen et al. 2025; Xiao et al. 2025a; Xu et al. 2025a). Furthermore, the two genera are phylogenetically distinct: *Dentisympodium* is nested within Pleurotheciaceae (Pleurotheciales, Savoryellomycetidae), whereas *Pseudodactylaria* belongs to Pseudodactylariaceae (Pseudodactylariales, Sordariomycetidae) (Hyde et al. 2024). Based on these combined morphological and molecular distinctions, *Dentisympodium* is here established as a new genus to accommodate this previously undescribed taxon.

Multi-locus phylogenetic analyses indicate that *Dentisympodium* forms a well-supported monophyletic clade, occupying a sister-group relationship with *Phragmocephala* within Pleurotheciaceae. Despite this close phylogenetic association, the two genera are morphologically and ecologically distinct: *Dentisympodium* produces hyaline conidia, whereas *Phragmocephala* is characterized by dematiaceous conidia (Hughes 1958; Réblová et al. 2016; Bao et al. 2022). Notably, molecular phylogenetic evidence demonstrates that *Phragmocephala* is polyphyletic, with species distributed across three families: Melanommataceae (Pleosporales), Microthyriaceae (Microthyriales), and Pleurotheciaceae (Pleurotheciales) (Su et al. 2015; Hernández-Restrepo et al. 2017; Bao et al. 2022). This underscores the critical role of molecular data in accurate fungal species delimitation and the resolution of complex taxonomic relationships.

The discovery of *Dentisympodium* further expands the diversity of lignicolous freshwater fungi in China, particularly in Guizhou Province. The description of this species contributes to refining the taxonomic framework of the family Pleurotheciaceae and provides a valuable reference for future surveys of freshwater fungi.

### Taxonomic key to the genera of Pleurotheciaceae

**Table:** 

1	Conidia helicoid	*** Helicoascotaiwania ***
–	Conidia not helicoid	**2**
2	Conidia muriform or dictyoseptate, with both transverse and longitudinal septa	**3**
–	Conidia aseptate or transversely septate only	**6**
3	Conidia globose to subglobose, dark brown to black	*** Neomonodictys ***
–	Conidia cylindrical, ellipsoidal, obclavate, pyriform or irregularly shaped	**4**
4	Conidia becoming fissitunicate at maturity	*** Rhexoacrodictys ***
–	Conidia not fissitunicate	**5**
5	Conidia pyriform to obclavate, muriform	*** Dematipyriforma ***
–	Conidia cylindrical to fusiform, with numerous transverse and longitudinal septa	*** Coleodictyospora ***
6	Conidiogenous cells denticulate and sympodially proliferating	**7**
–	Conidiogenous cells not denticulate-sympodial	**8**
7	Conidia solitary, acropleurogenous, 0–3-septate, mostly 3-septate, cylindrical, fusiform to obclavate, hyaline, smooth, prominently guttulate	*** Dentisympodium ***
–	Conidia solitary, cylindrical to obclavate, 0–1-septate, lacking conspicuous guttules	*** Pseudodactylaria ***
8	Conidia bearing distinct setulae	*** Anapleurothecium ***
–	Conidia lacking setulae	**9**
9	Conidia produced from phialidic conidiogenous cells	**10**
–	Conidia produced from non-phialidic conidiogenous cells	**12**
10	Conidia produced in slimy heads	*** Phaeoisaria ***
–	Conidia not produced in slimy heads	**11**
11	Conidiophores with terminal sterile extensions; conidia elongate	*** Pleurothecium ***
–	Conidiophores lacking sterile extensions; conidia short-cylindrical to ellipsoidal	*** Pleurotheciella ***
12	Conidia predominantly aseptate	**13**
–	Conidia septate	**15**
13	Conidia triangular to trigonous	* Melanotrigonum *
–	Conidia globose, ellipsoidal or obovoid	**14**
14	Conidia solitary	*** Monotosporella ***
–	Conidia catenate or aggregated	*** Pseudosaprodesmium ***
15	Conidiogenous cells phialidic with conspicuous sterigmata	*** Sterigmatobotrys ***
–	Conidiogenous cells lacking sterigmata	**16**
16	Conidia obclavate to cylindrical, distoseptate	*** Phragmocephala ***
–	Conidia euseptate	**17**
17	Conidia brown, thick-walled	*** Saprodesmium ***
–	Conidia hyaline to pale brown	**18**
18	Conidia dark brown to black, smooth, solitary	*** Nigrellomyces ***
–	Sexual morph dominant; ascospores versicolorous	*** Adelosphaeria ***

## Supplementary Material

XML Treatment for Dentisympodium

XML Treatment for Dentisympodium fusiforme

## Additional information

### Artificial Intelligence (AI) use

The authors accept full responsibility for the content of the manuscript, including the disclosure of any use of AI.

No AI tools were used in the preparation of this manuscript.

### Data availability

All of the data that support the findings of this study are available in the main text.
